# Bacterial content and associated risk factors influencing the quality of bulk tank milk collected from dairy cattle farms in Mandalay Region

**DOI:** 10.1002/fsn3.945

**Published:** 2019-02-11

**Authors:** Ye Wint Naing, Soe Soe Wai, Thant Nyi Lin, Wink Phyo Thu, Lat Lat Htun, Saw Bawm, Tin Tin Myaing

**Affiliations:** ^1^ Livestock Upgrading Section Livestock Breeding and Veterinary Department Mingalardon Myanmar; ^2^ Department of Veterinary Public Health University of Veterinary Science Yezin Myanmar; ^3^ Department of Pharmacology and Parasitology University of Veterinary Science Yezin Myanmar; ^4^ University of Veterinary Science Nay Pyi Taw Myanmar

**Keywords:** bulk tank milk, coliform count, milking cows, risk factors, total bacterial count

## Abstract

To investigate the bacterial content and risk factors associated with the hygienic quality of raw milk, a cross‐sectional study was conducted in four townships of Mandalay Region, Myanmar. From April to October 2017, bulk tank milk samples (*n* = 233) were collected from 233 dairy cattle farms located in Tada‐U, Pyin Oo Lwin, Meiktila, and Patheingyi Townships. From each farm, approximately 100 ml of bulk tank milk was collected and examined for bacterial content. Total bacterial count (TBC) and coliform count (CC) in milk samples were determined using milk agar and violet red bile agar. Of 233 milk samples, 68.2% (159/233) showed TBC higher than 1.0 × 105 cfu/ml, and 78.4% (183/233) showed CC higher than 100 cfu/ml. The mean value of TBC among 233 farms was 2.55 × 107 cfu/ml, ranging from 6.0 × 103 to 3.0 × 109 cfu/ml, whereas the mean value of CC was 1.59 × 105 cfu/ml, ranging from 10 to 8.4 × 106 cfu/ml. TBC tended to increase as CC increased in milk samples. The number of precautionary measures for milking operation, choice of cleaning materials, training experience of the farmers, cleanliness score of milking cows, and CMT scores of milk were significantly associated (*p* < 0.05) with TBC in bulk tank milk. Similarly, the number of precautionary measures for milking operation, choice of cleaning materials, training experience of the farmers, cleanliness scores of milking cows, CMT scores of milk samples, herd size, and type of milking practice showed significant association (*p* < 0.05) with CC in bulk tank milk. The effects of these potential risk factors should be minimized, farmers should be trained properly, and technical support should be provided, so that the quality of raw milk produced in Myanmar can be improved.

## 1 INTRODUCTION

Milk is one of the most valuable food regularly consumed among people. Due to high nutrient composition, milk production has been popular and played an important role in global food security. However, on the other hand, milk is highly vulnerable to bacterial contamination and can be the source of some food‐borne diseases (Jay, 2000). Raw milk can be contaminated with pathogens originated from dairy cows or farm environment. Bacteria can be transferred into milk during milking or at any stage of milk handling, through dirty udders, improperly sanitized milking equipment, and cows with subclinical mastitis (Amagliani et al., 2012; Kessel, Karns, Lombard, & Kopral, 2011).

Many milk‐borne human diseases are spread through the consumption of contaminated milk (Parekh & Subhash, 2008). Bacteriological safety of milk continues to be a topic of concern in the dairy industry and public health domain. In general, the microbial content of milk is a major factor that determines the quality of milk. In order to provide safe and healthy milk products, the Hazard Analysis and Critical Control Points (HACCP) system should be launched at every stage of milk collection, processing, and storage (Oliver, Boor, Murphy, & Murida, 2009).

Today, a variety of microbiological count methods, including the total bacterial count (TBC) and coliform count (CC), is available for monitoring the hygienic quality of raw milk (Jayarao, Pillal, Swant, Wolfgang, & Hedge, 2004). Among them, the TBC is the most common method used for evaluating the hygienic quality of raw milk, which estimates the total number of bacteria present in milk (Ruegg & Reinemann, 2002). The CC measures the number of coliform bacteria in milk primarily originating from cow's environment. The elevation of CC in milk is an indicator of poor sanitary practices in farm (Reinemann, Wolters, & Rasmuseen, 2000).

According to the report of Ministry of National Planning and Economic Development, Myanmar, the amount of milk produced in Myanmar has been increasing over years, which was 1,818 metric ton (MT) in 2013, 1,962 MT in 2014, 2,164 MT in 2015, and 2,375 MT in 2016, respectively. Despite the rise in production growth, milk and dairy products still have to be imported every year owing to increased consumption led by population growth. Livestock Breeding and Veterinary Department (LBVD) reported that the per capita consumption of milk was 26.7 kg in 2007, 28.57 kg in 2009, 31.71 kg in 2011, and 46.88 kg in 2015. Since 2014, the import of milk and dairy products has costed more than 100 million USD per year (LBVD, 2017).

In terms of the local industry, raw milk production is most elevated in Mandalay Region, where the dairy cattle population is highest. However, one major weakness in dairy industry of Myanmar is the lack of scientific information regarding the quality of raw milk. Although some research activities have been conducted on milk and milk products, the information about the quality of raw milk, such as total bacterial count and coliform count in milk, is still missing. Such information is crucial for the improvement and standardization of the quality of raw milk, as well as preventing the communicable food‐borne diseases. Therefore, this study was carried out with the objectives of determining the microbial content of bulk tank milk and identifying the risk factors associated with the increased microbial content in raw milk produced from dairy cattle farms in Mandalay Region of Myanmar.

## 2 MATERIALS AND METHODS

### 2.1 Study area

**Figure 1:**
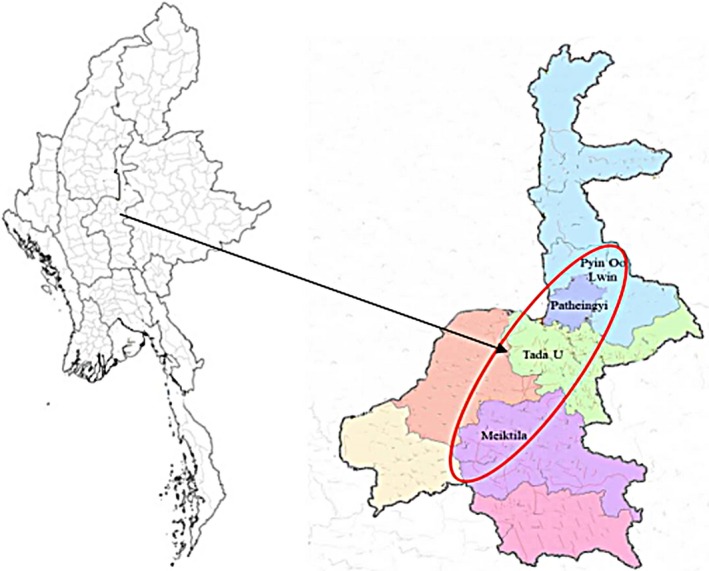
Map of study area

This study was conducted in four townships, namely Tada‐U, Pyin Oo Lwin, Meiktila, and Patheingyi townships, in Mandalay Region (Figure 1).

### 2.2 Sample size

The total number of dairy cattle farms in four townships was 4,422. Therefore, according to Daniel (1995), the number of farms to collect the bulk tank milk samples was calculated to be 233, by assuming that the prevalence is 80%, margin of error is 5, and confidence interval is 95%, respectively. The total sample size was then divided into four townships proportionate to the number of farms in each township (Table 1).

**Table 1:** Number of dairy cattle farms and milk sample collected from four townships

No.	Township	Targeted farms	Sampled farms
1	Tada‐U	3,126	163
2	Pyinoolwin	151	9
3	Meiktila	529	29
4	Patheingyi	616	32
	Total	4,422	233

### 2.3 Sample collection

A total of 233 milk samples were collected from 233 dairy cattle farms in four townships (one bottle of milk from collection tank [bulk tank milk] was taken as one sample unit). Prior to sample collection, the bulk tank milk was thoroughly mixed and stirred well to disperse the milk fat, using a sterile plunger. After homogenization, at least 100 ml of bulk tank milk sample was taken from the collection tank and transferred into the labeled bottle. Once taken, samples were immediately put in an ice box and transported to Mandalay Veterinary Diagnostic Laboratory (LBVD). The samples were then tested as described by Graham and Frank (2004).

### 2.4 Data collection

Information related to the background history, general condition, and management systems of the sampled farms was obtained through the questionnaires. Structural questionnaires designed with hypothesized risk factors that include (1) herd size (<30 vs. >30), (2) type of milking practice (hand vs. machine), (3) number of precautionary measures taken for milking operation (no operation vs. one operation vs. more than one operation), (4) choice of cleaning materials for washing the milking utensils (cold vs. hot vs. chlorine), and (5) training experience of the farmers (yes vs. no) were presented to the farm owners to check the association between the hypothesized risk factors and increased microbial content in milk, with reference to TBC and CC in sampled bulk tank milk.

### 2.5 Mastitis and cleanliness score classification

Following the questionnaire interview, further information regarding the mastitis and cleanliness score of the cows in sampled farms was recorded as described in 2.5.1 and 2.5.2.

#### 2.5.1 Cow cleanliness score

The hygiene of the cows was evaluated based on visual cleanliness scores adapted by Schreiner and Ruegg (2003). Evaluation was performed in four areas of each animal's body: the legs (L), flanks (F), abdomen (A), and the udder (U). Chronologically, score 1 (VC) indicates very clean, score 2 (C) indicates clean, score 3 (D) indicates dirty, and score 4 (VD) indicates very dirty. Farms were considered clean (Category 1) if the number of milking cows with the cleanliness scores of VC and C is equal to or more than 50% of total milking cows in the herd and taken as dirty (Category 2) if the number of milking cows with cleanliness scores of D and VD is equal to or more than 50% of total milking cows in the herd.

#### 2.5.2 California mastitis test (CMT)

The milk sample and CMT reagent are mixed in equal amounts, and the paddle was rotated. Depending on the gel formation, CMT scores were classified into five categories: negative (N), trace (T), weak positive (1), distinct positive (2), and strong positive (3). The reactions as strong as T (trace) or stronger than that (1, 2, and 3) were taken as positive, implying that the cow was suffering from subclinical mastitis or mastitis (Schalm & Noorlander, 1957). Based on the test results, the farms were divided into two groups, CMT‐positive group (Mastitis group) and CMT‐negative group (Nonmastitis group).

### 2.6 Bacteriological examination of bulk tank milk

Bulk tank milk samples were examined for TBC and CC. For determination of TBC, 10 ml of milk sample was added into the sterile tube containing 90 ml of peptone water. After thoroughly mixed, the concentration of the solution was serially diluted in 10‐fold proportion up to 10^−7^ concentration. Sample solution equal to or higher than 10^−3^ was transferred from the tubes to the plates, onto which 12–15 ml Difco™ milk agar was added later. For coliform count test, the same procedure was employed for 10‐fold serial dilution of the milk sample, as it was done in TBC. Unlike TBC, sample solution equal to or less than 10^−5^ concentration was transferred from the tubes to the plates, onto which 12–15 ml Difco™ violet red bile agar was added later. For both TBC and CC, mixing was done by rotating the plate in clockwise and anticlockwise directions for three or more times. The solidification of agar occurred within 10 min. Once solidified, plates for TBC were incubated at 32°C for 72 hr and those for CC at 32°C for 24 hr. Plate counts were expressed as the number of colony‐forming units per milliliter (cfu/ml) for both TBC and CC (National Conference on Interstate Milk Shipments (NCIMS), 2013).

### 2.7 Statistical analysis

The results from plate count tests on both TBC and CC were compared with the European Standard counts recommended by Regulation CE No. 852/853/854/2004, which is <1 × 10^5 ^cfu/ml for TBC and <100 cfu/ml for CC in milk sample. The association between the hypothesized risk factors and bacterial content of milk sample for both TBC and CC was analyzed by Pearson's chi‐square test using Statistical Package for Social Science (SPSS version 20). The association was considered significant, if *p* value less than 0.05 (*p* < 0.05) was observed between the hypothesized risk factor and bacterial content, either TBC or CC, of milk samples.

## 3 RESULTS

### 3.1 Farm characteristics

In this study, 233 farmers were interviewed regarding the background history, general condition, and management systems of the farms. Most of the farmers in the study area were smallholders. Demographic information about the type of milking practice (hand vs. machine), number of precautionary measures taken for milking operation (none vs. one vs. more than one), choice of cleaning materials for washing the milking utensils (cold vs. hot vs. chlorine), and training experience of the farmers was described in Table 2.

**Table 2:** Demographic data focused on management system in dairy cattle farms

No	Factors	Samples	Percentage (%)
1	Herd size		
	≤30	220	94.42
	>30	13	5.58
2	Milking type		
	Hand milking	229	98.28
	Machine milking	4	1.72
3	Cleaning of utensils		
	Cold water	183	78.54
	Hot water	21	9.01
	Chlorinated detergent	29	12.45
4	Precautionary measures		
	No operation	191	81.97
	One operation	33	14.16
	More than one operation	9	3.86
5	Training		
	Yes	59	25.3
	No	174	74.7

### 3.2 Overall total bacterial counts and coliform counts in bulk tank milk in four townships

**Figure 2:**
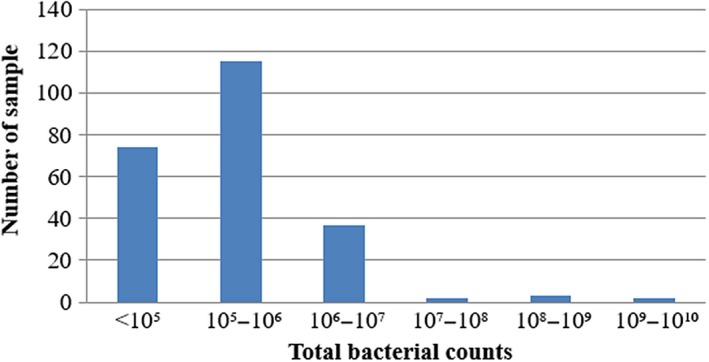
Overall occurrences of total bacterial counts in four townships

**Figure 3:**
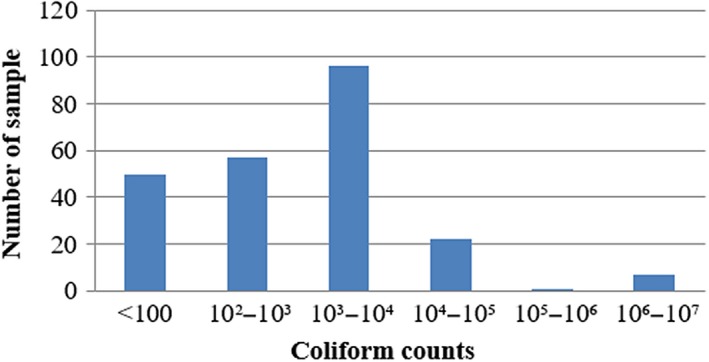
Overall occurrences of coliform counts in four townships

Out of 233 bulk tank milk samples, 68.24% (159 of 233) of bulk tank milk samples had TBC higher than 1.0 × 10^5 ^cfu/ml. The mean value of TBC was 2.55 × 10^7 ^cfu/ml, with the counts ranging from 6.0 × 10^3^ to 3.0 × 10^9 ^cfu/ml (Figure 2). Among the 233 samples analyzed, about 78.54% (183/233) of milk samples were found contaminated with coliform. The mean value of CC among four townships was 1.59 × 10^5 ^cfu/ml, with the counts ranging from the minimum of 10 cfu/ml to the maximum of 8.4 × 10^6 ^cfu/ml (Figure 3).

### 3.3 Total bacterial counts and coliform counts in bulk tank milk from each township

**Figure 4:**
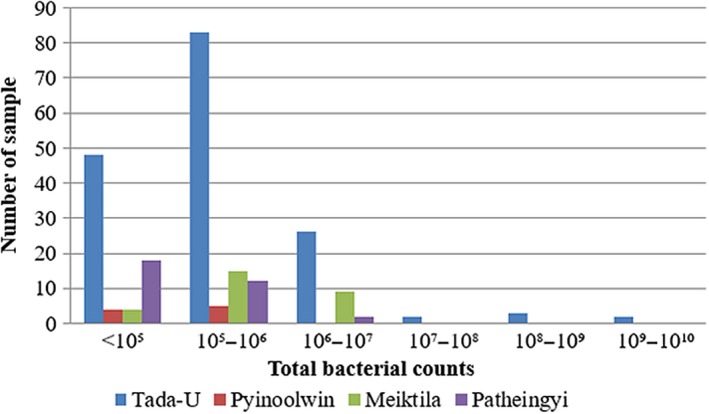
Occurrence of total bacterial counts in each township

The mean value of total bacterial counts was 3.6 × 10^7 ^cfu/ml (ranging from 6 × 10^3^ to 3.0 × 10^9 ^cfu/ml) in Tada‐U, 3.44 × 10^5 ^cfu/ml (ranging from 2.6 × 10^4^ to 8.9 × 10^5 ^cfu/ml) in Pyinoolwin, 8.15 × 10^5 ^cfu/ml (ranging from 7.2 × 10^4^ to 2.96 × 10^6 ^cfu/ml) in Meiktila, and 3.3 × 10^5 ^cfu/ml (ranging from 6.0 × 10^3^ to 5.4 × 10^6 ^cfu/ml) in Patheingyi Township, respectively (Figure 4).

**Figure 5:**
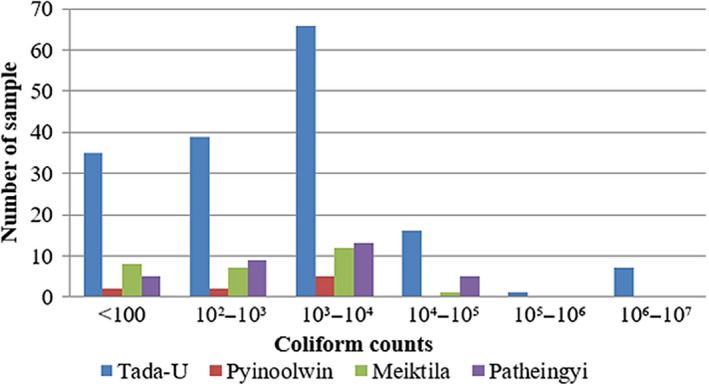
Occurrence of coliform counts in each township

The mean value of coliform counts was 2.25 × 10^5 ^cfu/ml (ranging from 20 to 8.4 × 10^6 ^cfu/ml) in Tada‐U, 1.61 × 10^3 ^cfu/ml (ranging from 40 to 4.8 × 10^3 ^cfu/ml) in Pyinoolwin, 2.71 × 10^3 ^cfu/ml (ranging from 10 to 2.7 × 10^4 ^cfu/ml) in Meiktila, and 3.3 × 10^5 ^cfu/ml (ranging from 20 to 7.1 × 10^4 ^cfu/ml) in Patheingyi Township, respectively (Figure 5).

### 3.4 Univariate analysis

Hypothesized risk factors were analyzed by Pearson's chi‐square test. The results showed that the number of precautionary measures taken for milking operation, choice of cleaning materials for washing the milking utensils, training experience of the farmers, cleanliness score of milking cows, and CMT score of milk samples produced significant association (*p* < 0.05) with total bacterial counts in bulk tank milk (Table 3). Similarly, the number of precautionary measures for milking operation, choice of cleaning materials, training experience of the farmers, cleanliness score of milking cows, CMT scores of milk samples, herd size, and type of milking practice showed significant association (*p* < 0.05) with CC in bulk tank milk (Table 4).

**Table 3:** Association between hypothesized risk factors and bacterial content (TBC) in milk

Factors	TBC		Mean	Occurrence (%)	*p* value	OR	95% CI	
	Good	Poor	(cfu/ml)				Lower	Upper
Herd size								
≤30	68	152	2.7 × 10^7^	69.1				
>30	6	7	3.62 × 10^5^	53.8	0.251	0.522	0.169	1.611
Milking type								
Hand	71	158	2.59 × 10^7^	68.9				
Machine	3	1	1.76 × 10^5^	25.0	0.061	0.150	0.015	1.465
Precautionary measures								
No	51	140	3.1 × 10^7^	73.3				
One	16	17	3.12 × 10^5^	51.5	0.012^a^	0.387	0.182	0.823
>One	7	2	1.2 × 10^5^	22.2	0.001^b^	0.104	0.021	0.517
Cleaning utensil								
Cold	46	137	3.2 × 10^7^	74.9				
Hot	8	13	5.19 × 10^5^	61.9	0.202	0.546	0.213	1.399
Chlorine	20	9	1.78 × 10^5^	31.0	0.000^b^	0.151	0.064	0.355
Training								
Yes	26	48	6.25 × 10^5^	55.9				
No	33	126	3.4 × 10^7^	72.4	0.019^a^	2.068	1.121	3.814
Cleanliness								
Clean	46	61	5.56 × 10^5^	57.0				
Dirty	28	98	4.60 × 10^7^	77.8	0.001^b^	2.639	1.495	4.659
CMT score								
Negative	43	31	2.59 × 10^5^	41.9				
Positive	31	128	3.75 × 10^7^	80.5	0.000^b^	5.727	3.125	10.498

*Notes*

CI: confidence interval; OR: odds ratio.

^a^Significantly; ^b^Highly significantly.

**Table 4:** Association between hypothesized risk factors and bacterial content (CC) in milk

Factors	CC		Mean	Occurrence (%)	*p* value	OR	95% CI	
	Good	Poor	cfu/ml				Lower	Upper
Herd size								
≤30	44	176	1.69 × 10^5^	80.0				
>30	6	7	3.69 × 10^3^	53.8	0.026^a^	0.292	0.093	0.911
Milking type								
Hand	46	183	1.63 × 10^5^	79.9				
Machine	4	0	70	0.00	0.002^b^	–	–	–
Precautionary measures								
No	26	165	1.95 × 10^5^	86.4				
One	17	16	1.31 × 10^3^	48.5	0.000^b^	0.148	0.067	0.329
>One	7	2	1.48 × 10^3^	22.2	0.000^b^	0.045	0.009	0.229
Cleaning utensil								
Cold	23	160	6.0 × 10^3^	87.4				
Hot	10	11	2.19 × 10^3^	52.4	0.000^b^	0.158	0.060	0.414
Chlorine	17	12	2.88 × 10^3^	41.4	0.000^b^	0.101	0.043	0.239
Training								
Yes	24	35	4.67 × 10^3^	59.3				
No	26	148	2.12 × 10^5^	82.4	0.000^b^	3.903	2.005	7.597
Cleanliness								
Clean	42	65	1.18 × 10^3^	60.7				
Dirty	8	118	2.89 × 10^5^	93.7	0.000^b^	9.531	4.221	21.519
CMT score								
Negative	25	49	8.4 × 10^4^	66.2				
Positive	25	134	2.34 × 10^5^	84.3	0.003^b^	2.735	1.437	5.206

*Notes*

CI: confidence interval; OR: odds ratio; –: could not calculated.

^a^Significantly; ^b^Highly significantly.

## 4 DISCUSSION

In this study, the mean value of TBC in bulk tank milk was 2.5 × 10^7 ^cfu/ml, with the maximum of 3.0 × 10^9 ^cfu/ml and the minimum of 6.0 × 10^3 ^cfu/ml. It was lower compared to the work of Khin Zar Lin (2015) conducted in Nay Pyi Taw, Myanmar, whose mean value for TBC in bulk tank milk was 2.67 × 10^8 ^cfu/ml. It was to note that this study focused on larger population of milking cows, compared to the stated study in Nay Pyi Taw, and, still, a lower mean value of TBC in bulk tank milk was observed in this study. This implied that the involvement of more commercial dairy farms, with better sanitary facilities in this study, reduced the impact of TBC on bulk tank milk.

However, despite the improvement, the TBC in this study was still higher than those reported from Malaysia 1.2 × 10^7 ^cfu/ml (Chye, Abdullah, & Ayob, 2003), United States 1.2 × 10^4 ^cfu/ml (Pantoja, Reinemann, & Ruegg, 2009), Canada 1.2 × 10^4 ^cfu/ml (Elmoslemany et al., 2010), and Albania 3.89 × 10^6 ^cfu/ml (Beli, 2015), indicating that the hygienic measures currently practiced in Myanmar need to be improved. Furthermore, the TBC in this study was higher than the standard value recommended by European Commission, 2004 (1.0 × 10^5 ^cfu/ml). Therefore, despite the presence of reports with higher TBC in bulk tank milk from some countries, such as 1.28 × 10^9 ^cfu/ml in Bangladesh (Uddin, Motazzim‐ul‐Haque, & Noor, 2011) and 3.8 × 10^7 ^cfu/ml in Iran (Rezaei et al., 2014), this study highlighted the need of good sanitation and better management in cow milk production of Myanmar.

In term of coliform count, the mean value of CC in this study was 1.59 × 10^5 ^cfu/ml, with the counts ranging from 10 to 8.4 × 10^6 ^cfu/ml. It was lower than the mean value of previous study conducted by Nang Khin Thuzar Aung (2016) in Nay Pyi Taw, Myanmar, whose mean value for CC in bulk tank milk was 2.05 × 10^9 ^cfu/ml. Similar to the case of TBC, the lower CC values in bulk tank milk in this study were attributed to the superior farm management system in commercial dairy farms compared to those of the small‐scale farms. However, the CC in this study was still higher than the CC of some reports, including 21 cfu/ml in Canada (Elmoslemany, Keefe, Dohoo, & Dingwell, 2009) and 4.7 × 10^2 ^cfu/ml in Bangladesh (Hasan, Islam, Mahmud, Uddin, & Ahmed, 2015). Again, the average CC values in this study exceed the standard value of 1.0 × 10^2 ^cfu/ml, recommended by European Commission, and therefore need to be improved if higher quality of milk is to be achieved.

Among the four townships, the highest mean value of TBC in bulk tank milk, 3.6 × 10^7 ^cfu/ml, was observed in Tada‐U Township, while the lowest, 3.3 × 10^5 ^cfu/ml, was detected in Patheingyi Township. The highest mean value of CC, 3.3 × 10^5 ^cfu/ml, was detected in Patheingyi Township, and the lowest (1.61 × 10^3^) was detected in Pyinoolwin Township. A possible reason to the elevation of TBC and CC in Tada‐U and Patheingyi Township could be due to poor hygienic condition of the collection tanks distributed by milk collectors. The collection tanks provided by the milk collectors were of poor hygienic condition in Tada‐U and Patheingyi Townships, while it was relatively clean in other two townships. The reason behind this can be different sanitary measures among various companies working as milk collector in four townships. It was to assume that bacteria deposited in the collection tanks multiplied over time and became a major source of contamination, particularly in the absence of regular cleansing (Reinemann et al., 2000).

In this study, the risk factors for the increased TBC and CC in milk were very similar. The number of precautionary measures taken for milking operation, choice of cleaning materials for milking utensils, training experience of the farmers, cow cleanliness score, and CMT score of milk sample were significantly associated (*p* < 0.05) with the increase of both TBC and CC in bulk tank milk. Basically, the contamination of milk with bacteria starts at the farm level, where the production of milk originates. There were studies reporting that the microbial contamination of raw milk at the farm level could be associated with several risk factors, such as improper cleanliness of the farms, poor sanitizing procedures of the milking equipment, and storage of milking utensils (Kelly et al., 2009; Murphy & Boor, 2000).

In agreement with the report of Jayarao et al. (2004), this study revealed that the TBC in bulk tank milk was not influenced by the size of the herd (*p* > 0.05). For CC, the result of this study was in contrast to some studies which mentioned the larger herd size as a risk factor for increased CC in bulk tank milk (Elmoslemany et al., 2010; Goldberg et al., 1992; Gran, Mutukumira, Wetlesen, & Narvhus, 2002; Jayarao et al., 2004). Instead, this study revealed the larger herd size as a protective factor, in which the CC decreased as the herd size increased. It can be partly due to wide variation in the herd sizes of the farms in four townships. Unlike developed countries, herd size distribution is very uneven in this study. It can be as small as four or five milking cows to as large as hundreds of milking cows. There are very few commercial farms in Myanmar, and generally, commercial farms are bigger in herd size, facilitated with clean equipment, and bacterial contamination can be relatively low with them. Another point is that the owners of commercial farm are affordable of healthcare service and have their farms regularly checked for diseases.

From this study, it was realized that the farms that practice hand‐milking system were more likely to develop contamination than the machine‐milking farms. Though not significant, the mean value of TBC in milk collected from the machine‐milking farms (1.76 × 10^5 ^cfu/ml), almost the same with EU standard, was very low compared to the TBC (2.59 × 10^7 ^cfu/ml) of hand‐milking farms. For CC, it was significantly lower in the machine‐milking farms (70 vs. 1.63 × 10^5 ^cfu/ml). However, there was a previous report with contradictory statement in Myanmar, which described that no significant association existed between CC of bulk tank milk and type of milking system (Nang Khin Thuzar Aung, 2016).

On another point, out of 233 farms, up to 75% (3/4) of machine‐milking farms showed TBC <1.0 × 10^5 ^cfu/ml, while only 31% (71/229) of hand‐milking farms showed the same. Milking men could be the primary source of bacterial spread among farms, since they do not practice hand washing while moving from one farm to another. This finding agreed with the previous reports which described the milking men as the primary cause of contamination in bulk tank milk among farms (Filipovic & Kokaj, 2009; Poutrel et al., 2015).

The benefits of precautionary measure taken for milking operations, mostly forestripping, predipping, and postdipping methods, have been described as the principles to follow to reduce bacterial contamination during milking (Bade, Reinemann, & Thompson, 2008; Jayarao et al., 2004; Murphy & Boor, 2000; Ruegg, 2003). In this study, among the 233 dairy farms, 33 farms performed any one of these three operations, while nine farms performed the combination of more than one operation. However, 191 dairy farms did not carry out any of these three operations at all. Farms that accomplished a milking operation, either one or more of the operations (fore‐stripping, pre‐dipping and post‐dipping), showed significantly lower TBC (2.16 × 10^5^ vs. 3.1 × 10^7 ^cfu/ml) and CC (1.39 × 10^3^ vs. 1.95 × 10^5 ^cfu/ml) compared to the farms that did not practice any of these methods. However, the difference between the farms that practiced only one method and those that practiced more than one method regarding the TBC and CC in bulk tank milk was very slight. This suggested that any one of these operations was adequate to reduce bacterial content in bulk tank milk.

Choice of cleansing agents for washing the milking utensils and their impact on bacterial content in bulk tank milk were also analyzed. About 78.5% of the farmers used cold water for washing, while 9.01% (21/233) used hot water and the other 12.4% (29/233) used chlorinated water. For TBC, milk from chlorinated water group was significantly lower than the other two groups. Reinemann et al. (2000) and Wallace (2009) also reported that the effective use of chlorine during milking process can reduce the TBC in milk. However, no significant difference (*p* < 0.05) was observed between the hot‐ and cold‐water groups regarding the TBC in bulk tank milk. It can be explained that some bacteria species, such as Micrococci and Bacilli, can survive heating at 63°C for 30 min, and *Enterococcus faecalis*, lactobacilli, and some corynebacteria are also heat‐resistant, surviving at 60°C for 20 min (Chambers, 2002). Unlike TBC, the CC in bulk tank milk was significantly lower in hot‐water groups, compared to the cold‐water group. This finding was supported by several studies indicating that the cold water as a potential source of microorganisms in milk, particularly when the disinfection process was inadequate (Jayarao et al., 2004; Murphy & Boor, 2000; Perkins et al., 2009). Likewise, Bava et al. (2011) and Gran et al. (2002) also claimed that the use of chlorinated water, as well as hot water, would significantly reduce the numbers of microorganisms in bulk tank milk.

It has been recognized that the knowledge of the farmers is important in dealing with the problem, and dairy farms need training for good hygiene strategies if higher milk production is to be achieved. Out of 233 respondents, nearly 25% of the respondents had been involved in training on hygienic milk production, while the rest, 75% of the farmers, had not been involved in such trainings. It is noteworthy that a significant difference of TBC and CC existed between trained and untrained farms, indicating that the knowledge on hygienic measures had led to better quality of milk. This study was in agreement with Melin (2015) who concluded that the training had positive effect on the quality of milk.

In this study, milking cows were mostly kept under unclean environmental condition and more than 50% of milking cows remained dirty at the time of milking. Agreed with the previous studies by Elmoslemany et al. (2009) and Zucali et al. (2011), a strong association was observed between increased bacterial content in bulk tank milk and cleanliness score of the milking cows. The TBC was 2.5 times higher and CC was 9.5 times higher in the dirty milking‐cow group than in the clean milking‐cow group. This finding suggested the dirty udder and teats as the important source of bacterial contamination in milk, which could exceed 10,000 cfu/ml in very dirty cows (Wallace, 2009). Pankey, Wildman, Drechsler, and Hogan (1987) and Saxena and Rai (2013) also reported that the amount of dirt on the teats prior to milking was positively associated with increased TBC in bulk tank milk. Similarly, the dirty cows were 1.5 times more likely to be infected with major mastitis pathogen when compared to clean milking cows (Schreiner & Ruegg, 2003).

With both TBC and CC higher in milk samples with CMT‐positive results, the CMT scores of milk samples were found significantly associated with increased bacterial content in bulk tank milk. This finding was consistent with the previous works of Jayarao et al. (2004) and Pantoja et al. (2009), who reported a small to moderate correlation between mastitis and increased microbial count in the milk of dairy cows. Pandey and Voskuil (2011) reported that the milk secreted from a healthy udder contained only a very few bacteria, approximately 500–1,000 cfu/ml. Wallace (2009) also revealed that mastitis cows could shed microorganisms as much as 10,000,000 cfu/ml, and mastitis‐causing bacteria were believed to be potential contaminants of bulk tank milk (Hayes et al., 2001; Zadoks, Gonzalez, Boor, & Schukeen, 2004).

Finally, this study revealed that the microbial content of milk produced by local farmers was fairly high and needed to be improved. The quality of milk deteriorated by elevated TBC and CC was a pure public health concern. It was clearly seen that the production of good quality milk was hampered by the high microbial content and associated risk factors. To bring the quality of raw milk to a satisfactory level, farmers should be aware of the risk factors influencing the quality of milk and should be able to control them. To date, there are no microbial standards available for the assessment of the quality of raw milk produced in Myanmar. In this context, a series of tests and modifications for the quality control of raw milk are deemed necessary. Innovative approach, such as quality‐based payment system, should be adopted to encourage for the improvement of raw milk quality. Scientific studies and research activities are also in need. Taken all these together, it will be of great benefits to the livelihoods of the farmers, food safety of the consumers, and sustainability of dairy industry in Myanmar, if the production of high‐quality milk can be achieved.

## CONFLICT OF INTEREST

The authors declare that there is no conflict of interest regarding the publication of this article.

## ETHICAL APPROVAL

For ethical statement, the procedures and protocols used in sample collection and laboratory analysis of this study were approved by the research administrative board of Livestock Breeding and Veterinary Department (LBVD) and the ethical committee of post‐graduate research under the University of Veterinary Science (UVS).
